# Bronchial Tree System Analysis of Live Beluga Whale (*Delphinapterus leucas*) Using Bronchoscopy

**DOI:** 10.3390/vetsci11010033

**Published:** 2024-01-15

**Authors:** Takashi Kamio, Yukako Odani, Wataru Ohtomo, Akira Ogushi, Yuichiro Akune, Masanori Kurita, Ayaka Okada, Yasuo Inoshima

**Affiliations:** 1 Port of Nagoya Public Aquarium, 1-3 Minato-machi, Minato-ku, Nagoya 455-0033, Japan; 2 Laboratory of Food and Environmental Hygiene, Cooperative Department of Veterinary Medicine, Gifu University, 1-1 Yanagido, Gifu 501-1193, Japan; 3 Joint Graduate School of Veterinary Sciences, Gifu University, 1-1 Yanagido, Gifu 501-1193, Japan

**Keywords:** beluga whale, bronchial tree system, bronchoscopy, *Delphinapterus leucas*

## Abstract

**Simple Summary:**

Cetaceans, including beluga whales (*Delphinapterus leucas*), have high morbidity and mortality rates due to bacterial or fungal lower respiratory infections. Bronchoalveolar lavage fluid (BALF) collection by bronchoscopy is beneficial for detecting pathogenic microorganisms in the lower respiratory tract. In this study, bronchoscopy was performed on five captive beluga whales (9–44 years old) to detect bronchial length and bifurcation. The lengths from the blowhole to the scope impassable points due to the minimized bronchi diameters of the left principal bronchus (LPB), right principal bronchus (RPB), and tracheal bronchus (TB) were 110–155, 110–150, and 80–110 cm. The observed bifurcated bronchi of the LPB, RPB, and TB were more than 10, 10, and 6 bifurcated bronchi. Unique differences of bifurcated bronchi for each individual were observed in LPB, RPB, and TB. These results could be useful for obtaining BALF to diagnose lower respiratory infections in beluga whales.

**Abstract:**

Cetaceans, including beluga whales (*Delphinapterus leucas*), have high morbidity and mortality rates due to bacterial or fungal lower respiratory infections. Bronchoalveolar lavage fluid (BALF) collection by bronchoscopy is beneficial for detecting pathogenic microorganisms in the lower respiratory tract. Efficient and safe bronchoscopy requires characterizing the bronchial tree systems of beluga whales, as no reports exist on bronchial length and bifurcation. In this study, bronchoscopy was performed on five captive beluga whales (9–44 years old) to detect bronchial length and bifurcation. The lengths from the blowhole to the scope impassable points due to the minimized bronchi diameters of the left principal bronchus (LPB), right principal bronchus (RPB), and tracheal bronchus (TB) were 110–155, 110–150, and 80–110 cm, respectively, and were correlated with the body length. Bronchoscopy identified more than 10, 10, and 6 bifurcated bronchi from the LPB, RPB, and TB, respectively. This is the first report to clarify the differences in bronchial tree systems between beluga whales and other cetaceans, as well as the differences for each individual beluga whale. These results could be useful for obtaining BALF via bronchoscopy to detect pathogenic microorganisms causing infections in the lower respiratory tract of beluga whales.

## 1. Introduction

Respiratory infections caused by bacteria and fungi are significant causes of morbidity and mortality in wild and captive cetaceans, including beluga whales (*Delphinapterus leucas*) [1,2,3,4,5]. Early detection of respiratory diseases in cetaceans is essential for successful treatment, as several respiratory diseases are life-threatening [2,3,4,6,7]. In humans, examination of the bronchoalveolar lavage fluid (BALF) obtained using a bronchoscope is beneficial for detecting pathogenic microorganisms that may be present in the lower respiratory tract [8]. Although bronchoscopy is becoming commonplace in bottlenose dolphins [5,9], it has not yet been established in beluga whales, including BALF collection. Exhalation is unreliable for detecting pathogenic microorganisms in the lower respiratory tract [2,7]. While thoracic radiography can evaluate the airway, lung surface, and deep lung tissue, it cannot detect pathogenic microorganisms [10].

Anatomical approaches for the respiratory systems of cetaceans, including beluga whales, have been described previously [6,11,12,13]. The bronchial tree system of cetaceans comprises three bronchi: the left principal bronchus (LPB, *bronchus principalis dexter*), right principal bronchus (RPB, *bronchus principalis sinister*), and tracheal bronchus (TB, *bronchus trachealis*) [6,12,14,15,16,17]. TB, which bifurcates from the trachea anterior into LPB and RPB, is a specific structure for cetaceans and terrestrial Artiodactyls [6,12,14,15,16,17]. Previous studies have reported guidelines for performing cetacean bronchoscopy [5,9]. However, little was reported about the standardized method of performing BALF in beluga whales [5]. In cetaceans, several studies for upper respiratory tracts have been reported [6,12,14,15,16,17]. Limited studies for lower respiratory tracts, such as harbor porpoises (*Phocoena phocoena*) [6], Ganges River dolphins (*Platanista gangetica*) [15], and striped dolphins (*Stenella coeruleoalba*) [16], have been reported, but not for beluga whales.

In beluga whales, reports about respiratory physiology were described previously [11,18,19]. However, no study has been reported concerning their bronchial tree systems, such as the lengths from the blowhole to the scope impassable points due to the minimized bronchi diameters (impassable points) or the number and site of the bifurcated bronchi. Furthermore, the available information is insufficient to enable an appropriate bronchoscopy procedure. Therefore, identifying the bronchial tree system of beluga whales is essential to perform bronchoscopy accurately. In this study, we aimed to identify the number and sites of bifurcated bronchi and the length from the blowhole to the impassable points of the LPB, RPB, and TB for a practical understanding of bronchial mucosa observations and BALF collection.

## 2. Materials and Methods

### 2.1. Animals

The bronchial tree systems of five beluga whales (three males and two females, 9–44 years old) in the managed care in the Port of Nagoya Public Aquarium (PNPA, Nagoya, Japan) were examined using bronchoscopy (Table 1). Bronchoscopies were performed following a routine examination. The animal experimental procedures were reviewed and approved by the Gifu University Animal Care and Use Committee (approval number 2020-264).

### 2.2. Sedation

Bronchoscopies were conducted thrice for each beluga whale under sedation. A combination of midazolam [0.023–0.122 mg/kg intramuscular injection (I.M.), KS6769, Sandoz, Tokyo, Japan] and butorphanol (0.023–0.061 mg/kg I.M., VETLI5, Meiji Seika Pharma Co., Ltd., Tokyo, Japan) was administered 30 min prior to bronchoscopy in all cases except 25 August 2021 on DL-6 [Diazepam (EB011, Takeda Pharmaceutical Co., Ltd., Osaka, Japan) and tramadol (Pfizer, Tokyo, Japan) per os 120 min prior to bronchoscopy] [5,20,21]. Butorphanol was administered to alleviate discomfort [20]. Sedative medicines caused respiratory depression; however, the respiratory rate remained stable with the appropriate doses. Doses of midazolam and butorphanol were prescribed by following their body weights and ages and adjusted by detailed observations, as described previously [22]. Flumazenil (0.0020–0.0059 mg/kg I.M., AH21A, Fuji Pharma Co., Ltd., Toyama, Japan) was used as a reversal agent when necessary [20,22].

**Table 1:** Bronchoscopic observations of beluga whales (*Delphinapterus leucas*).

ID	Date of Birth	Sex	Examined Date	Body Length (cm)	Body Weight (kg)	Length from Blowhole to Impassable Points (cm)			Number of Observed Bronchi		
						LPB	RPB	TB	LPB	RPB	TB
DL-1	1978 Expected ^(1)^	Male	24 August 2021	371	686	130	128	100	11	10	6
			21 October	371	673	132	130	100	11	10	6
			19 November	371	672	130	125	100	11	10	6
DL-6	1995 Expected ^(2)^	Female	25 August 2021	407	808	125	130	100	10	11	6
			15 November	407	769	140	122	100	10	11	6
			20 December	407	788	135	135	95	10	11	6
DL-9	25 July 2007	Female	27 October 2021	360	610	120	115	85	12	12	6
			25 February 2022	360	651	115	115	85	12	12	6
			13 June	360	574	110	110	80	12	12	6
DL-11	2 August 2012	Male	8 November 2021	355	508	113	115	83	12	11	7
			26 March 2022	355	520	120	115	90	12	11	7
			11 October	360	491	113	115	85	12	11	7
DL-12	2007 Expected ^(2)^	Male	11 December 2021	450	1179	155	150	110	12	12	9
			30 May 2022	454	1255	145	145	105	12	12	9
			30 January 2023	455	1187	140	140	105	12	12	9

Their ages were expected by ^(1)^ the growth layer groups in the thin section of tooth [23], or ^(2)^ their body lengths [24]. LPB: left principal bronchus; RPB: right principal bronchus; TB: tracheal bronchus.

### 2.3. Bronchoscopy

Bronchoscopy was performed using endoscopes with diameters of 9.4 and 9.3 mm (EN-450T5/W, Fujifilm, Tokyo, Japan, and VQ-9303C, Olympus, Tokyo, Japan), following the procedure used in previous studies on bottlenose dolphins [5,9]. Ethanol was chosen as a disinfectant because it has been classified as one of the intermediate-level disinfectants for proceeding with bronchoscopy [25]. The entire procedure was performed aseptically, in accordance with the plan for the scope to reach the lower respiratory tract [5]. The nomenclature for the bronchial tree system of the beluga whale followed that of harbor porpoises, as described previously [6]. The first, second, and third lobar branches of the LPB and RPB were identified as left bronchus 1 (LB1) and right bronchus 1 (RB1), LB2 and RB2, and LB3 and RB3, respectively [6,26]. The first, second, and third lobar branches of the TB were identified as tracheal bronchus 1 (TB1), TB2, and TB3, respectively [6,26].

The bronchoscopic image and the animal body position were adjusted in advance [5]. The scope was inserted through the epiglottis [5,9]. To alleviate discomfort, 4 mL of 2% lidocaine (614435304, Sandoz Pharma Co., Ltd., Tokyo, Japan) was sprayed onto the tracheal mucosa immediately after insertion of the scope past the epiglottis [5,9]. The scope could not reach the terminal bronchi (diameter: 0.9 mm) due to the thickness of the scope [11]. The bronchial tree systems of the LPB, RPB, and TB were evaluated from the entrance to the impassable points by observing the bifurcated bronchi while taking a video. To determine the necessary length of scopes enough to observe the bronchi mucosa and obtain BALF from the impassable points of the bronchi, the length from the blowhole to each impassable point of the LPB, RPB, and TB was measured using the inserted scope length. The scope was manipulated at the center of the bronchi to minimize damage to the bronchial mucosa as the position of the scope changed due to their breathing. The correlation between the bronchi and body length of the animals was calculated via least-squares regression, which included the estimation of R^2^ values, to conduct trend analyses.

### 2.4. Diagrammatic Dorsal View of the Bronchial Tree

Bronchoscopy videos were recorded thrice for each of the five beluga whales. Unique diagrammatic dorsal views of the bronchial trees were illustrated by identifying the branching patterns of the segmental bronchi.

## 3. Results

### 3.1. Bronchoscopy

**Figure 1:**
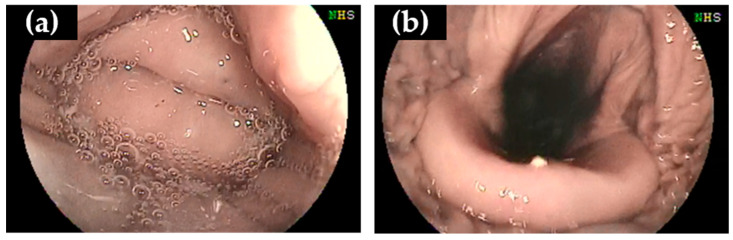
Bronchoscopy images of the beluga whale (*Delphinapterus leucas*) (DL-1). The epiglottis closed (**a**) and opened (**b**) during the respiratory cycle.

Each beluga whale was administered the appropriate sedation dosage for bronchoscopy. The respiratory rate of the whales decreased when the scope was fixed in front of the epiglottis and inserted into the trachea. The epiglottis was visualized when the scope was inserted to a depth of 25–30 cm from the blowhole. The epiglottis shut tightly (Figure 1a) and opened for a limited time during breathing (Figure 1b), which was similar to that described for bottlenose dolphins [9]; the endoscope was quickly inserted through the epiglottis during breathing.

**Figure 2:**
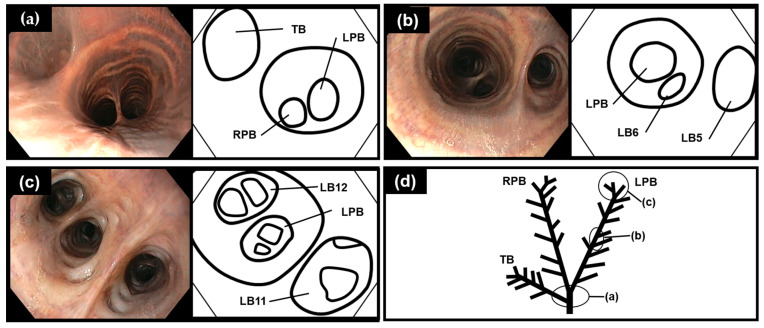
Monopodial and dichotomous-like bronchial bifurcation in the beluga whale (*Delphinapterus leucas*) (DL-11). (**a**) Bifurcation of the left and right principal bronchus (LPB and RPB) and tracheal bronchus (TB). (**b**) Monopodial bifurcation in the upper parts of the lower respiratory tract. (**c**) Monopodial and dichotomous-like bifurcation near the impassable points of the lower respiratory tract. (**d**) Simple drawing of bronchial tree system with points at the level of the images of (**a**–**c**). LB5 and 6: left bronchus 5 and 6; LB11 and 12: left bronchus 11 and 12.

The entrances of the LPB, RPB, and TB were visualized when the scope was inserted to a depth of 60–70 cm from the blowhole (Figure 2a). The entrance of the TB was observed cranial to the carina (Figure 2a). The RPB was nearly co-linear with the trachea, whereas the LPB originated at a slightly more acute angle from the trachea (Figure 2a). The bronchial bifurcations of the beluga whale were monopodial in the upper parts of the lower respiratory tract (Figure 2b); however, monopodial and dichotomous-like bifurcations were observed close to the impassable points of the lower respiratory tract (Figure 2c). The diameters of the LPB, RPB, and TB were larger than their bifurcated bronchi (Figure 2b) until LB8, RB8, and RT5. The diameters of the bifurcated bronchi at close to the impassable points were similar to that of LPB, RPB, and TB (Figure 2c). Bronchoscopy revealed 10–12, 10–12, and 6–9 bifurcated bronchi in the LPB, RPB, and TB, respectively (Table 1). Equal numbers of bifurcated bronchi were observed using 9.3 and 9.4 mm-diameter scopes.

**Figure 3:**
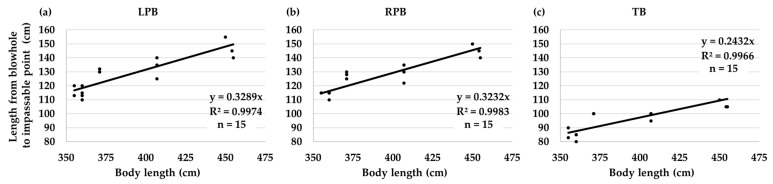
The lengths from the blowhole to the impassable points due to the minimized bronchi diameters of the left principal bronchus (LPB) (**a**), right principal bronchus (RPB) (**b**), and tracheal bronchus (TB) (**c**) show correspondence to the body length of all beluga whales (*Delphinapterus leucas*) by using 9.3–9.4 mm diameter scopes.

The lengths from the blowhole to the impassable points of the LPB, RPB, and TB were 110–155, 110–150, and 80–110 cm, respectively (Table 1). These lengths corresponded to the body length of the beluga whales (Figure 3). Differences were observed in the bronchial length of the same beluga whale (Table 1), which could be due to the scope’s bend.

### 3.2. Diagrammatic Dorsal View of the Bronchial Tree Systems

**Figure 4:**
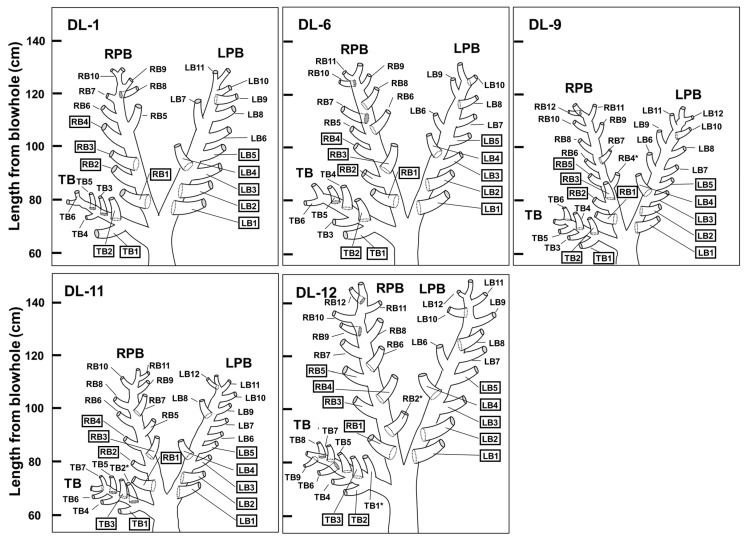
Diagrammatic dorsal view of the unique bronchial tree systems. Right bronchus 4 (RB4) in DL-9, tracheal bronchus 2 (TB2) in DL-11, and RB2 and TB1 in DL-12 may have been irregularly observed (asterisk). The left bronchus (LB) 1–5, RB1–4, and TB1–2 in DL-1 and DL-6 bifurcated from globally similar sites (square) in five beluga whales (*Delphinapterus leucas*). The left and right principal bronchi (LPB and RPB) and TB are displayed.

Diagrammatic dorsal views of the unique bronchial tree systems of the five beluga whales are illustrated based on bronchoscopic visualizations (Figure 4). The nomenclature used by Amis and McKiernan [26] was easily applied to the tracheal bronchi, and it has been reported to be applicable to harbor porpoises [6]. Bifurcated bronchi were denoted in a numeric sequence from the proximal to distal position according to their order of appearance from each principal bronchus [6].

## 4. Discussion

In the context of bronchoscopy, this study found that the effective lengths of scopes for observation and BALF collection can be estimated by their body lengths (Figure 3). The correlation between body lengths and bronchi lengths was expected because the total lung capacity can be estimated from the body mass in beluga whales [18], the same as in terrestrial animals [11]. In bottlenose dolphins, scopes with tube diameters of 8–9 mm can reach a depth of approximately 70–80 cm, whereas scopes with a tube diameter of 3 mm can reach a depth of approximately 90–110 cm [5]. This study clarified that 160 cm effective lengths were enough to reach each the impassable points of LPB, RPB, and TB with a 9.3–9.4-mm diameter scope for beluga whales with up to 455 cm body length (Table 1). As for bottlenose dolphins, scopes longer than 160 cm will be required to reach the impassable points when narrower diameter scopes are inserted for beluga whales. Bronchoscopies for beluga whales are easier with trachea and bronchi diameters wider than for bottlenose dolphins; however, one needs to go deeper.

Bifurcated bronchi directions from LPB, RPB, and TB in beluga whales were classified as dorsal, ventral, medial, and lateral to clarify the unique characteristics. The numbers of bifurcated bronchi were 4–5 to dorsal, none to ventral, 0–3 to medial, and 3–7 to lateral in LPB, 2–4 to dorsal, 0–2 to ventral, 1–3 to medial, and 4–5 to lateral in RPB, and 1–3 to dorsal, 0–2 to ventral, 0–1 to medial, and 2–3 to lateral in TB (Figure 4). These results clarify that most bifurcated bronchi from the LPB, RPB, and TB are directed dorsally and laterally. In contrast, a minority of them are directed medially and ventrally in all bronchial tree systems in five beluga whales. Despite the differences in bifurcated bronchi numbers directed to each side, total counts of bifurcated bronchi from LPB, RPB, and TB showed few differences (Table 1 and Figure 4). These results reveal that bronchial tree systems in beluga whales exhibit dislocation, absence, and addition, as described previously in horses [27].

In all bronchial tree systems of the five beluga whales, LB1 and LB2 bifurcated dorsally, LB3 bifurcated laterally, LB4 bifurcated dorsally, LB5 bifurcated laterally, and RB1 bifurcated dorsally (Figure 4). RB4 (bifurcated medially) in DL-9; TB2 (bifurcated ventrally) in DL-11; and RB2 (bifurcated dorsally), TB1 (bifurcated medially) in DL-12 may have been irregularly added, as these bifurcated bronchi exist only in one out of five beluga whales. Without these bifurcated bronchi, the directions of RB1–RB4 and TB1–TB2 in DL-1 and DL-6 were the same in all five beluga whales. However, due to the complicated dislocation, absence, and addition of bifurcated bronchi, the construction of a standard bronchial tree system was not completed in this study.

The tracheal structures of beluga whales were found to be similar to other cetaceans [6,12,14,15,16,17]. TB was observed cranial to the bifurcation to LPB and RPB [6,12,14,15,16,17]. The body lengths of beluga whales corresponded to their bronchi lengths but not to the numbers and angles of bifurcated bronchi (Table 1 and Figure 4). Differences in bronchial tree systems of beluga whales born in the wild (DL-1, DL-6, and DL-12) and those in managed care (DL-9 and DL-11, respectively) were compared to clarify the environmental effects of bronchial tree systems; however, no significant differences were observed (Table 1 and Figure 4). Additionally, differences in bronchial tree systems of same-age beluga whales, DL-9 (female) and DL-12 (male), were compared (Table 1 and Figure 4); however, they were not clarified due to the difference in their body lengths (Table 1). Differences in bronchial tree systems among ages were compared to clarify the aging effects on bronchial tree systems. Beluga whales were classified as old (DL-1), adults (DL-6), and young adults (DL-9, DL-11, and DL-12) (Table 1). Additional bifurcated bronchi were observed only from beluga whales classified as young adults; however, due to the limited cases, the relationship between bronchial tree systems and ages was not clarified (Figure 4). Unique variations among beluga whales were similar to those among horses [27]; however, unique variations among other cetaceans have not been reported due to limited cases [6,15,16]. Additional research has the potential to detect unique variations among the same species in other cetaceans, such as beluga whales.

To clarify the difference in bronchial tree systems between beluga whales and other cetaceans, the directions of bifurcated bronchi from LPB and RPB in beluga whales were compared to harbor porpoises [6], Ganges River dolphins [15], and striped dolphins [16]. In harbor porpoises and striped dolphins, the majority of bifurcated bronchi were directed laterally, whereas a minority of them were directed dorsally and medially, and no bifurcated bronchi ventrally [6,16]. Additionally, four bronchial tree systems of harbor porpoises exhibited few anatomical differences [6]. In Ganges River dolphins, the majority of bifurcated bronchi were directed laterally and dorsally, whereas a minority of them were directed ventrally, and no bifurcated bronchi were directed medially [15]. In this study, the bronchial tree systems of beluga whales had several bifurcated bronchi directed ventrally and medially (Figure 4). These results clarify that the bronchial tree systems of beluga whales have differences from those of other cetaceans [6,15,16].

Nomenclatures of bronchial tree systems for cetaceans were established to permit serial evaluations of bronchial mucosa lesions or secretions and appropriate communication between veterinarians about the lesions [6,27]. Despite the lack of a standard bronchial tree system due to unique variations among beluga whales, understanding each beluga whale’s specific bronchial tree system can facilitate appropriate diagnosis and progress observation.

## 5. Conclusions

This study revealed that bronchial tree systems exhibit differences between cetacean species. Bronchial tree systems research for each cetacean species should be conducted. This is the first report to clarify the differences in bronchial tree systems between beluga whales and other cetaceans, as well as the differences for each individual beluga whale. These results could be useful for obtaining BALF via bronchoscopy to detect bronchial mucosa conditions, pathogenic microorganisms, and inflammatory signs.

## Data Availability

The data presented in this study are available within the article.
